# The Effect of Low Dose Sildenafil On Verapamil - Induced Cardiovascular Toxicity in Rats

**DOI:** 10.1186/2197-425X-3-S1-A500

**Published:** 2015-10-01

**Authors:** F Gul, NC Duman, MK Arslantas, M Haliloglu, I Cinel, MZ Gören

**Affiliations:** Marmara University, Department of Anaesthesiology, Division of Intensive Care Unit, Istanbul, Turkey; Marmara University, Department of Medical Pharmacology, Istanbul, Turkey

## Introduction

Experimental studies have shown that sildenafil, a phosphodiesterase type 5 inhibitor, may have significant cardioprotective effects if used in low doses [[1, 2]].

## Objectives

The aim of this study was to compare the efficacies of glucagon and sildenafil treatments in anesthetized rats receiving verapamil overdose.

## Methods

Male Sprague-Dawley rats (n = 8 per group), weighing 300-350 g were used in the study. the iliac arteries and veins of rats bilaterally were catheterized under urethane anaesthesia. Upon completion of catheterization, the rats were connected to a monitor to observe blood pressure and heart rate throughout the procedures. Toxicity was induced by infusion of verapamil 15 mg/kg until 10 minutes. the rats received either continuous infusion of sildenafil (0.06 mg/kg/h), glucagon (2mg/kg/h) or 0.3mcg/kg/min noradrenaline added to sildenafil 0.06 mg/kg/h infusion. a naive controls and saline treated control experiments were also done. the rats were sacrificed by cervical dislocation at 60 min. in order to make an integrative comparison, the area under curve in the pressure vs time plot of each rat was calculated. One-way analysis of variance was used followed by Tukey’s test for statistical analysis.

## Results

Verapamil infusion produced decreases in the mean arterial pressure values (MAP). the AUC_10-60 min_ of MAP plot was significantly lower in the control group, and both treatments produced significant increases in the AUC_10-60 min_ of MAP plots (p < 0.05). Noradrenaline added to the sildenafil infusion was found to generate better values in the cardiovascular parameters (p < 0.01).

Comparison of pulse pressure did not yield a significant difference. Heart rate values were restored better in the group that received noradrenaline adjunct.

## Conclusions

The results of this study may imply that sildenafil alone is no better than glucagon, the classical treatment approach in verapamil toxicity. However, sildenafil may be more effective if used with a vasopressor adjunct.

**Figure 1:**
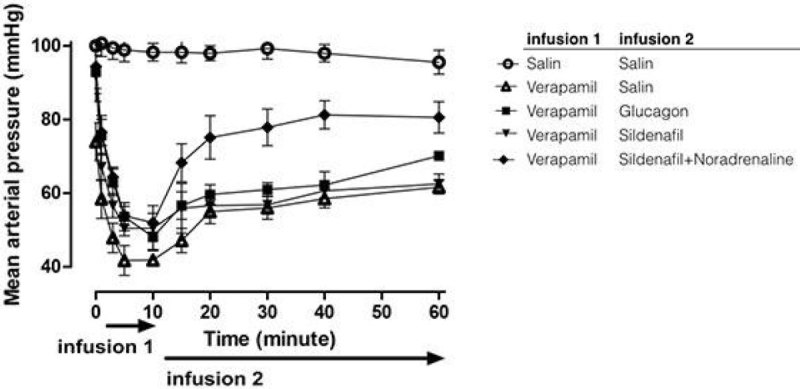
***The MAP (mean ± SD) of the rats during verapamil intoxication according to treatment groups.***
